# Chromosome-scale genome assembly of medicinal plant *Tinospora sagittata* (Oliv.) Gagnep. from the Menispermaceae family

**DOI:** 10.1038/s41597-024-03315-y

**Published:** 2024-06-12

**Authors:** Mohammad Murtaza Alami, Shaohua Shu, Sanbo Liu, Zhen Ouyang, Yipeng Zhang, Meijia Lv, Yonghui Sang, Dalin Gong, Guozheng Yang, Shengqiu Feng, Zhinan Mei, De-Yu Xie, Xuekui Wang

**Affiliations:** 1 College of Plant Science and Technology, Huazhong Agricultural University, Wuhan, China; 2 China Resources Sanjiu (Huangshi) Pharmaceutical Co., Ltd., Huangshi, 435000 Hubei China; 3 Department of Plant and Microbial Biology, North Carolina State University, Raleigh, NC 27695 USA

**Keywords:** Genome assembly algorithms, Molecular engineering in plants

## Abstract

*Tinospora sagittata* (Oliv.) Gagnep. is an important medicinal tetraploid plant in the Menispermaceae family. Its tuber, Radix Tinosporae, used in traditional Chinese medicine, is rich in diterpenoids and benzylisoquinoline alkaloids (BIAs). To enhance our understanding of medicinal compounds’ biosynthesis and Menispermaceae’s evolution, we herein report assembling a high-quality chromosome-scale genome with both PacBio HiFi and Illumina sequencing technologies. PacBio Sequel II generated 2.5 million circular consensus sequencing (CCS) reads, and a hybrid assembly strategy with Illumina sequencing resulted in 4483 contigs. The assembled genome size was 2.33 Gb, consisting of 4070 scaffolds (N50 = 42.06 Mb), of which 92.05% were assigned to 26 pseudochromosomes. *T. sagittata*’s chromosomal-scale genome assembly, the first species in Menispermaceae, aids Menispermaceae evolution and *T. sagittata*’s secondary metabolites biosynthesis understanding.

## Background & Summary

*Tinospora sagittata* is a perennial medicinal tetraploid (2n = 4X = 52) plant used in traditional Chinese medicine (TCM). It was officially listed with a Chinese medicine name, “Jin Guo Lan,” in the 2015 edition of the *Chinese Pharmacopoeia*^1^. Columbamine^2^, jatrorrhizine^3^, and palmatine^4^ are the three main medicinal BIAs in *T. sagittata*. In addition, other active compounds isolated from this plant include flavonoids^5^, lignans^6^, and clerodane type of diterpenoids^7^. These alkaloids from *T. sagittata* tuber have antifouling^8^, anti-inflammatory^9^, and α-glucosidase inhibitory activities^8^.

Moreover, these alkaloids, flavonoids, lignans, terpenoids, and other compounds provide multiple therapeutic uses of Radix Tinosporae in TCM. These include improvement of immune capacity, prevention against upper respiratory infections and lower oral ulcers, treatment of diabetes, anti-cancer properties, and protection of the liver from different diseases^10^. In addition to medicinal uses, *Tinospora* is a major group of angiosperms^11^, and *T. sagittata* is a model plant for studying species’ evolutionary relationships within the Menispermaceae family. It plays an important role in understanding the phylogenetic placement of the Menispermaceae family in flowering plants.

Herein, to enhance the knowledge related to the genome features of *T. sagittata*, we report genome and transcriptome assembly with different sequencing technologies. We assembled a high-quality genome and several transcriptomes that allowed for characterizing the phylogenetic placement of *T. sagittata* in the Menispermaceae family and the divergence time of this family in Ranunculales. The analysis revealed a monoploid genome size of approximately 553.23 Mb and a whole-genome size of 2.33 Gb, with a 2.98% heterozygosity. Despite the heterozygosity-challenging de novo assembly, the final assembly included 4,328,940 biallelic heterozygous sites across 26 chromosomes. PacBio Sequel II generated 2.5 million CCS reads, and a hybrid assembly strategy with Illumina sequencing resulted in 4483 contigs. The assembly was further improved using Hi-C, producing 4070 scaffolds and chromosome-scale sequences. Quality assessments, including BUSCO and CEGMA analyses, indicated high accuracy and completeness. The genome annotation identified 52,953 protein-coding genes using homology-based, Ab-initio-based, and RNAseq-based methods. Repetitive elements constituted 51.72% of the genome, with retroelements and long terminal repeats predominant. A high-quality genome of *T. sagittata* was assembled via short read (Illumina Hiseq) sequencing, long read sequencing (PabBio HiFi), and Hi-C sequencing. The genome features high heterozygosity and polyploidy. The assembled genome unearthed an ancient WGD event in *T. sagittata*, which was likely related to the divergence of Menispermaceae Papaveraceae and Ranunculaceae.

## Methods

### Plant materials

**Fig. 1:**
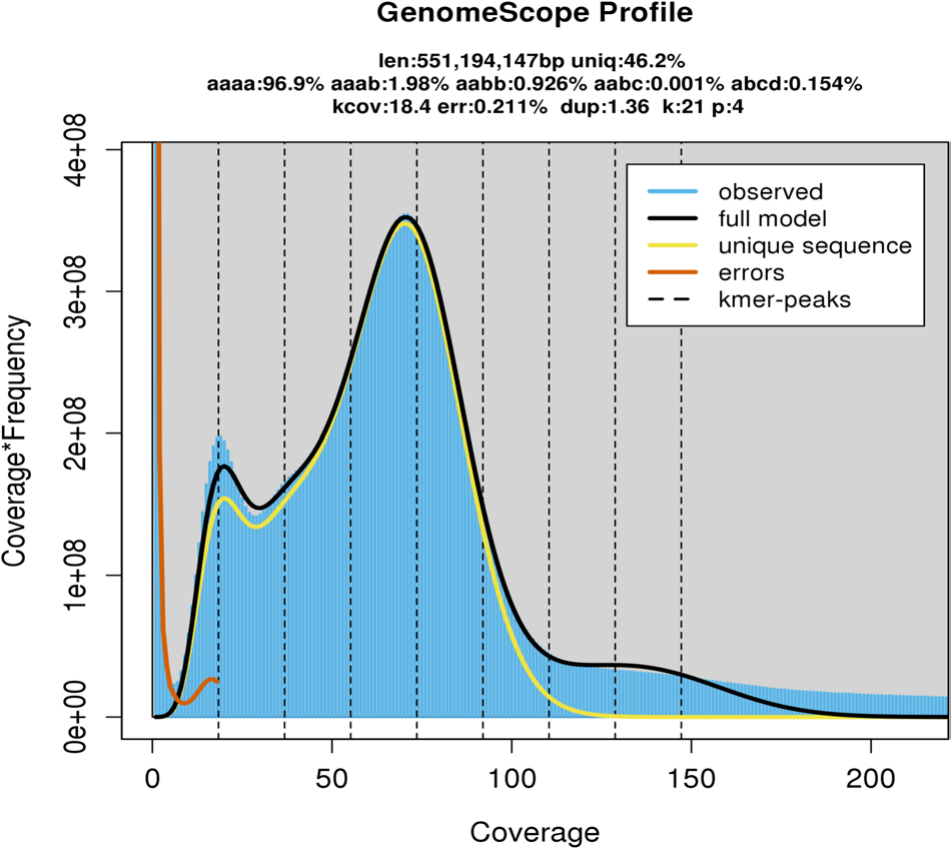
*21-mer* depth distribution of sequencing reads.

*Tinospora sagittata* is a medicinal tetraploid (2n = 4X = 52) plant that is cultivated for the production of rhizomes, namely Radix Tinosporae (RT, *Jinguolan* in Chinese) (Fig. 1A). Seeds were collected from Lichuan of Hubei Province, China, one of the areas of RT production. Seeds were planted in a controlled plant growth chamber with 22–25 °C, a relative humidity of 60–70%, and a 16-hour light/8-hour dark photoperiod. The light intensity was approximately 200 μmol/m^2^/s. After four months of seed germination, when seedlings reached about 20 cm high, they were planted in the research station at Huazhong Agricultural University and managed to keep away from pests. Young and healthy leaves were collected and washed with ultrapure water three times. The washed leaves were immediately frozen in liquid nitrogen and stored at −80 °C before DNA extraction. In addition, young and mature leaves were collected for gene expression profiling experiments and metabolomics, as described below.

### Genome size estimation

The size of the *T. sagittata* genome was estimated using a k-mer (k = 21) analysis-based approach and Illumina PE short reads. The Jellyfish (v2.1.4) software^12^ was used to count k-mer in the DNA sample. The GenomeScope^13^ software was used to estimate the genome size. A 21-mer analysis of the sequenced genome revealed that allotetraploid *T. sagittata* had a monoploid genome size of ~553.23 Mb and a whole-genome size of 2.33 Gb. The genome’s k-mer distribution displays three distinct peaks, potentially indicative of heterozygous, homozygous, and repeated k-mers. This analysis indicated that the genome of *T. sagittata* was characterized with a 2.98% heterozygosity (Fig. 1).

### Genome sequencing with PacBio technology

Genomic DNA was extracted from fresh leaves using the DNAsecure Plant Kit (TIANGEN), which followed the manufacturer’s protocol. The high-quality DNA samples were sheared to 10 kb in size for amplification according to Megaruptor® DNA Shearing System (PacBio, CA, USA). According to the manufacturer’s instructions, at least 10 μg of sheared DNA was used to construct SMRTbell libraries using SMRTbell Express Template Prep Kit 2.0 (PacBio, CA, USA). In brief, the steps include DNA concentration, damage repair, end repair, ligation of hairpin adapters, and template purification. The resulting SMRTbell libraries with an insert size of 60 kb were sequenced using the P6 polymerase/C4 chemistry combination on the PacBio Sequel platform (Pacific Biosciences, USA) according to the manufacturer’s protocol.

Preparing Hi-C libraries from fresh leaves followed a standard procedure as reported previously^14^. Five main steps are as follows: (1) cell cross-linking: fixing the samples with formaldehyde, cross-linking intracellular protein and DNA, preserving their interaction, and maintaining the 3D structure in the cell; (2) endonuclease digestion: using HindIII to digest DNA to produce sticky ends on both sides of the cross-link; (3) end repair: using an end repairing to introduce biotin-labeled bases to facilitate subsequent DNA purification and capture; (4) circularization: circularizing the DNA after end repairing and then circularizing the DNA fragments containing interactions to ensure that the position of the interacting DNA is determined during subsequent sequencing and analysis; and (5) DNA purification and capture: decrosslinking the DNA, purifying the DNA, fragmenting it into 300–700 bp fragments, and using streptavidin magnetic beads to capture the DNA fragments containing the interaction relationship for library construction. After the library was constructed, the concentration and insert size (300 bp) of the library were detected using Qubit2.0 and Agilent 2100, respectively, and the effective concentration of the library was accurately quantified using a Q-PCR method to ensure the quality of the library. The Illumina platform was used for high-throughput sequencing, and the sequencing read length was paired-end (PE) 150.

### Genome assembly

*De novo* assembly of sequences followed this pipeline. First, the long reads (60 kb) from the PacBio SMRT Sequencer were assembled using FALCON (https://github.com/PacificBiosciences/FALCON/)^15^. The longest subreads were selected as seed reads to correct sequence errors. Second, error-corrected reads were aligned to each other and assembled into genomic contigs using the following parameters: length-cutoff-pr = 10,000, max-diff = 95, and max-cov = 105. All genomic contigs were polished according to Quiver^16^. Third, based on the Illumina sequencing reads, tools of Pilon^17^ were used to correct errors. Fourth, sequences from the Hi-C sequencing were aligned to the assembled scaffolds according to BWA-MEM^18^. Finally, the scaffolds were clustered onto chromosomes according to LACHESIS (http://shendurelab.github.io/LACHESIS/)^19^. The sequencing with the PacBio Sequel II yielded 2.5 million CCS reads (average length 15.7 kb) with a total data volume of 43 Gb. The PacBio long reads were corrected and assembled with a hybrid assembly strategy before using 11.25 Gb of Illumina sequencing (short reads) for polishing (Figs. S1 & 2, Tables S1–4). The assembly of short reads from the Illumina sequencing and PacBio long reads resulted in 4483 contigs (N50 = 8.3Mbp) with a total of 1299.6 Mbp, in which the maximum length was 31.9Mbp and the GC content was 36.36% (Table 1).

**Table 1:** Statistical summary of the genome assembly of *T. sagittata*.

Data source	PacBio + Illumina	PacBio + Hi-C
Total scaffolds		1,299,652,234
Number of scaffolds		4,070
Scaffold N50(bp)		42,067,207
Scaffold N90(bp)		66,947
Scaffold Max		63,028,082
Total contigs Length	1,142,509,881	1,299,610,934
Number of contigs	2,531	4,483
Contigs N50(bp)	23,237,671	8,349,146
Contigs N90(bp)	1,234,648	65,341
Contigs Max	49,008,001	31,913,866
GC%	36.49	36.36

**Fig. 2:**
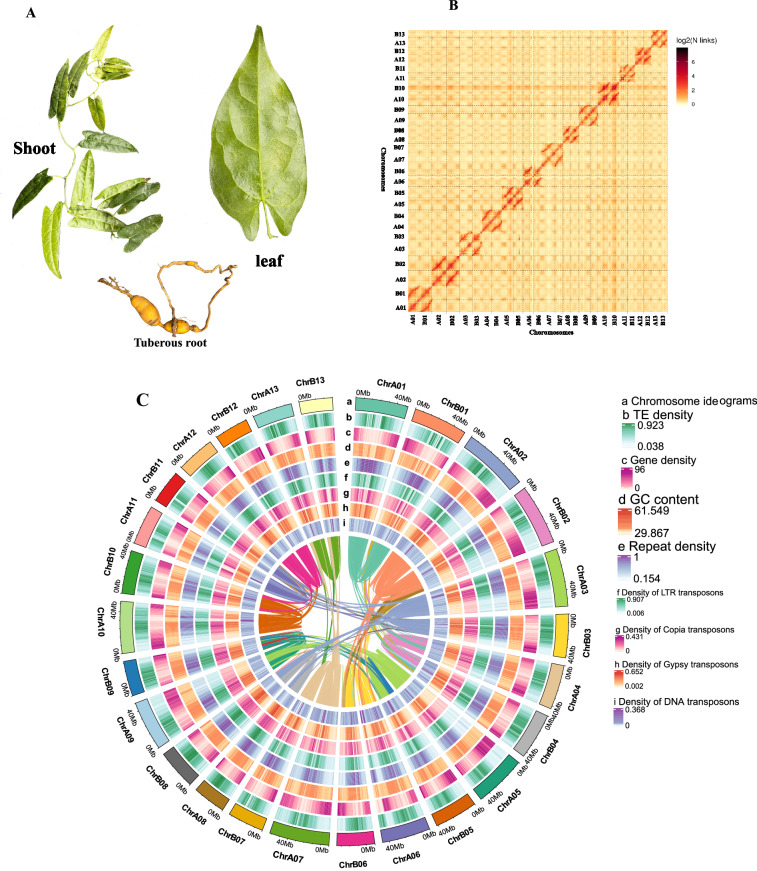
Main features of genome assembly of *T. sagittata*. (**A**) Three types of tissues of *T. sagittata* were collected from a research station at Huazhong Agricultural University, Hubei Province, China. (**B**) An interaction heat map of Hi-C assembled chromosomes. (**C**) Distribution features of the assembled genome are shown by four types of elements arranged from outer (a) to inner (i), (a) chromosome ideograms, (b) transposon elements (TE) density, (c) gene density, (d) GC content, (e) repeat density, (f) density of LTR elements, (g) density Copia transposons, (h) density of Gypsy transposons, and (i) density of DNA transposons.

After polishing PacBio long reads with Illumina short reads, the assembly was further scaffolded with Hi-C. The scaffolding results obtained 4070 scaffolds (N50 = 42.06 Mb). Subsequently, 960.8 million reads with 287,909.2 Mbp clean Hi-C paired-end reads were used for scaffold extension and anchoring. The Hi-C assembly and manual adjustment of the heatmap obtained 1,196.2 Mbp of genomic sequences, accounting for 92.05%, which were used for mapping to the 26 chromosomes. The results showed that 1,132.3 Mbp out of 1,196.2 Mbp (accounting for 94.66%) were mapped to the 26 chromosome sequences. A further sequence analysis obtained 572.7 Mb of reads uniquely aligned to the genome. In these unique sequences, 399.5 Mb (accounting for 69.75% of the uniquely aligned reads) were valid Hi-C data visualized with a heatmap (Fig. 2B, Table 1, Tables S5, S6). The 26 chromosomes were clearly distinguished in the heatmap to form 4070 unique groups. In each group, the intensity of the interaction at its diagonal position was higher than that at the non-diagonal position, indicating that those chromosomes assembled by Hi-C were adjacent to each other. The heat map also showed that the interaction signal strength between the sequences at the diagonal position) were strong, while that between non-adjacent sequences at off-diagonal positions was weak (Fig. 2B, Fig. S3). This result is consistent with the principle of Hi-C-assisted genome assembly. Based on the assembly of the chromosome-based genome, the analysis with the Circlize software provided chromosome ideograms, transposon elements (TE) density, gene density, GC content, repeat density, density of LTR elements, density Copia transposons, density of Gypsy transposons, density of DNA transposons and collinearity between the chromosomes (Fig. 2Ca–i).

### Genes annotation

We annotated gene functions using homology-based, de novo, and transcriptome-based predictions. First, homolog proteins from four plant genomes (*Arabidopsis thaliana*, *Coptis chinensis* Franch, *Macleaya cordata*, and *Aquilegia coerulea*) were downloaded from Ensemble Plants (http://plants.ensembl.org/index.html). Protein sequences from these genomes were aligned to the *T. sagittata* genome using TblastN^20^ with an *E*-value cutoff of 1e^−5^. The BLAST hits were conjoined with the Solar software^21^. GeneWise^22^ was used to predict the exact gene structure of the corresponding genomic regions for each BLAST hit (Homo-set). Second, for transcriptome-based prediction, RNA-seq data were mapped to the assembled genome using TopHat (version 2.0.8)^23^ and Cufflinks (version 2.1.1)^24^, and then the transcripts were assembled into gene models (Cufflinks-set). Third, RNA-seq data were assembled with Trinity^25^ and then used to create several pseudo-ESTs. These pseudo-ESTs were also mapped to the assembled genome, and PASA-predicted gene models were predicted using PASA^26^. Five ab initio gene prediction programs, AUGUSTUS (version 2.5.5)^27^, GenScan (version 1.0)^28^, GlimmerHMM (version 3.0.1)^29^, GeneID^30^, and SNAP^31^, were used to predict coding regions in the repeat-masked genome. Finally, gene model evidence from the Homo set, Cufflinks-set, PASA-T-set, and ab initio programs were combined via EvidenceModeler (EVM)^32^ to obtain a non-redundant set of gene structures. BLASTP^33^ (with an E-value cutoff of 1e^−5^) was performed via two integrated protein sequence databases: SwissProt (https://web.expasy.org/docs/swiss-prot_guideline.html) and NR. Protein domains were annotated by searching against the InterPro (V32.0)^34^ and Pfam (V27.0)^35^ databases using InterProScan (V4.8)^36^ and HMMER (V3.1)^37^ were used to predict the function of protein-coding genes. Gene Ontology (GO) terms were obtained from the corresponding InterPro or Pfam entry. BLAST assigned genes likely involved in the biosynthesis of the secondary metabolite against the KEGG databases with an E-value cutoff of 1e^−5^. Genes encoding tRNA were identified with the tRNAscan-SE software^38^. The rRNA fragments were predicted by aligning transcripts to the rRNA sequences using BlastN with an E-value cutoff of 1e^−10^. Those cDNAs encoding miRNA and snRNA were predicted with INFERNAL software^39^ against the Rfam database (release 9.1)^40^.

Homology-based, Ab-initio-based, and RNAseq-based methods were used to predict protein-coding genes. After removing theoretical nonfunctional genes, 52,953 protein-coding genes were obtained from the assembled genome (Table S7). Among the predicted genes, 1047, 3788, and 10 were unique in homology-based, Ab-initio-based, and RNAseq-based, respectively (Fig. S4). Tissue-specific RNA-seq was completed to develop transcriptomes. The resulting data showed that the average length of coding sequence genes was 6203.59 bp. The average coding sequence (CDS) length was 1360.42 bp, with an average of five exons and four introns per gene (Table 2). Approximately 97.93% of the genes were functionally annotated, of which 96.91% and 97.79% had significant hits in the NR and TrEMBL databases, respectively. Gene Ontology terms classified 82.91% of the genes. KEGG pathways annotated 75.21% of the genes (Table S8). These results indicate the high accuracy of the gene predictions in the *T. sagittata* genome. We further annotated noncoding RNA, yielding 9,624 transfer RNA genes, 13,014 ribosomal RNA genes, 350 small nuclear RNA genes, and 292 microRNA genes, as well as 287 pseudogenes in the *T. sagittata* genome (Tables S9–11). Next, we combined RNA-seq and full-length transcriptome data from four tissues and organs (leaf, rhizome, roots, and stem) with three biological replicates. At least 6.36 Gb of clean data were generated for each sample, with a minimum of 94.02% clean data, achieving a quality score of Q30. Clean reads of each sample were mapped to a specified reference genome. The mapping ratio ranged from 89.11% to 93.96% (Table S12).

**Table 2:** Primary statistical results of gene structure prediction of *T. sagittata* and relative species.

	Species				
	*Macleaya cordata*	*Tinospora sagittata*	*Coptis Chinensis*	*Arabidopsis thaliana*	*Aquilegia coerulea*
Gene Number	21911	52953	40011	27336	24823
Gene length	81601254	328498623	142726621	60260578	88406260
Average Gene length	3724.21	6203.59	3567.18	2204.44	3561.47
Exon Length	27578610	80970630	45674196	40495463	38484951
Average Exon Length	1258.67	1529.1	1141.54	1481.4	1550.37
Exon Number	116170	292255	194300	145214	130163
Average Exon Number	5.3	5.52	4.86	5.31	5.24
CDS Length	27578610	72038511	38570982	33328107	30437061
Average CDS length	1258.67	1360.42	964.01	1219.2	1226.16
CDS Number	116170	285953	184042	140233	124022
Average CDS Number	5.3	5.4	4.6	5.13	5
Intron Length	54022644	247527993	97052425	19765115	49921309
Average Intron Length	2465.55	4674.48	2425.64	723.04	2011.09
Intron Number	94259	239302	154289	117878	105340
Average Intron Number	4.3	4.52	3.86	4.31	4.24

### Repeat regions prediction

**Fig. 3:**
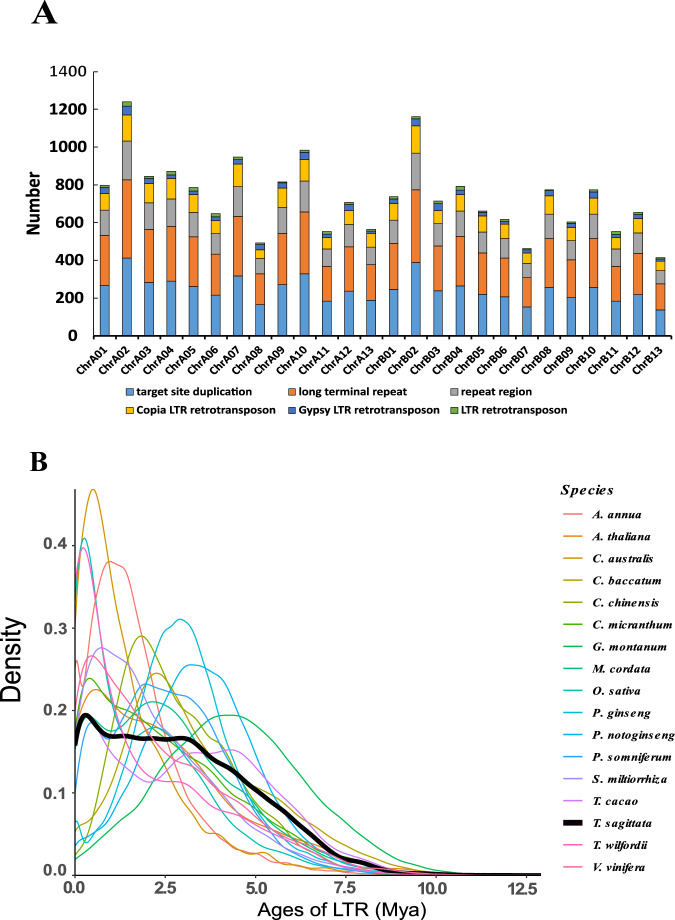
The distribution of insertion time of intact LTRs in *T. sagittata*. (**A**) Genomic constituents of LTR. The column represents the number. (**B**) Temporal patterns of LTR-RT insertion bursts in *T. sagittata*, compared with those in other 16 species.

Transposable elements (TEs) in the *T. sagittata* genome were searched by combining de novo-based and homology-based approaches. The de novo approach was completed with the RepeatModeler (http://www.repeatmasker.org/RepeatModeler/), LTR_FINDER (http://tlife.fudan.edu.cn/ltr_finder/), and RepeatScout (http://www.repeatmasker.org/) software to build a repeat library. The homology-based approach was carried out with the RepeatMasker (version 3.3.0) (http://www.repeatmasker.org/) software against the Repbase TE library and RepeatProteinMask (http://www.repeatmasker.org/) software against the TE protein database. Tandem repeats were detected in the genome using the Tandem Repeats Finder (TRF) software^41^. A total of 1,119,004 (51.72%) reads with a length of 672.2 Mb of the assembly were masked and annotated as repetitive elements (Figs. 2Ce-I, 3A), of which 45.13% was retroelement, while 6.59% was DNA transposon. Long terminal repeat (LTR) accounted for 41.85% of the repetitive elements, and long interspersed nuclear elements (LINE) were 2.92%. Interestingly, most LTRs were *Gypsy* and *Copy* elements (constituting 18.55% and 14.39% of the *T. sagittata* genome), and 8.32% comprised unknown LTR repeats (Table S13). About 91 Mb of tandem repeats were obtained, accounting for 7% of the genome (Table S14). The repeated element in the *T. sagittata* genome has experienced continuing amplification from 2 Mya (Fig. 3B).

## Data Records

The data supporting the findings of this work are available within the paper and its Supplementary Information files. Sequencing reads for *T. sagittata* are available on the NCBI Sequence Read Archive (SRA) https://identifiers.org/ncbi/insdc.sra: SRR28788848^42^ for genome survey data; SRR28790574^42^ for Hi-C data; and SRR27194311-SRR27194322^42^ for RNA sequencing data. Genome assembly for *T. sagittata* is available on GenBank https://identifiers.org/ncbi/insdc.gca:GCA_035771175.1^43^. Additionally, the genome annotation file and the gene family construction data were accessible on the figshare database^44^.

## Technical Validation

The quality of the drafted genome was evaluated with five tools. First, the high-quality reads from short insert-size PE libraries were mapped to the scaffolds using BWA-MEM. Second, to assess the completeness of the genome assembly, the obtained unigenes from *T. sagittata* transcriptome data were mapped to the scaffolds using BLAT^45^. Third, the Core Eukaryotic Genes Mapping Approach (CEGMA)^46^ pipeline was used to assess the completeness of the genome assembly or annotations. Finally, based on evolutionarily informed expectations of gene content from near-universal single-copy orthologues selected from OrthoDB v9^47^, benchmarking Universal Single-Copy Orthologues (BUSCO)^48^ analysis was performed to assess genome assembly, gene set, and transcriptome completeness. The Illumina short reads were aligned to the assembled genome using BWA^49^ to evaluate the assembly quality. The results revealed that the mapping rate of Illumina and PacBio sequencing was about 99.27%. Then, based on plant gene models, Benchmarking Universal Single-Copy Orthologs (BUSCO) analysis was completed to assess the assembled genome quantitatively. The results indicated that 96.78% of the BUSCO sequences were present in the *T. sagittata* genome, while only 0.99% and 2.23% were fragmented and missing, respectively (Table 3). Furthermore, Core Eukaryotic Genes Mapping Approach (CEGMA) analysis^46^ was completed to understand core protein-encoding orthologs. The resulting data disclosed that of 458 core eukaryotic genes (CEG), 421 (about 91.92% of CEGMA) were presenting in the assembled *T. sagittata* genome. In addition, of the 248 highly conserved CEGs, 190 (about 76.61%) existed in the assembled genome (Table 4).

**Table 3:** Assessment of the gene coverage rate using BUSCO.

	PacBio + Illumina		PacBio + Illumina + HiC	
Complete Single-Copy BUSCOs	968	59.98%	451	27.94%
Complete Duplicated BUSCOs	611	37.86%	1111	68.84%
Fragmented BUSCOs	12	0.74%	16	0.99%
Missing BUSCOs	23	1.43%	36	2.23%
Total BUSCO groups searched	1579	97.83%	1562	96.78%

**Table 4:** Assessment of the gene coverage rate using CEGMA.

	complete	complete + partial		
	# Prots	%Completeness	# Prots	%Completeness
PacBio + Illumina	423	92.36	186	75
PacBio + Illumina + HiC	421	91.92	190	76.61

### Supplementary information

Supplementary Figures

Supplementary Data

Supplementary Tables

## Data Availability

The study utilized freely available software to the public, and the parameters are explicitly outlined in the Methods section. In cases where specific parameters were not explicitly stated for the software, default settings recommended by the developers were employed. The study did not utilize custom scripts or code.
