# Supplementary material for: Chromosome-scale genome assembly of medicinal plant Tinospora sagittata (Oliv.) Gagnep. from the Menispermaceae family
Source: Sci Data. 2024 Jun 12;11:610. doi: 10.1038/s41597-024-03315-y (PMC11169364; doi:10.1038/s41597-024-03315-y)
Supplement: Supplementary file 1 — Supplementary Figures [file 41597_2024_3315_MOESM1_ESM.docx]

**Supplementary information**

**Title:** **Chromosome-scale genome assembly of medicinal plant *Tinospora sagittata* (Oliv.) Gagnep. from the Menispermaceae family**

Mohammad Murtaza Alami**^1^**, Shaohua Shu**^1^**, Sanbo Liu**^2^**, Zhen Ouyang**^1^**, Yipeng Zhang**^1^**, Meijia Lv**^1^**, Yonghui Sang**^1^**, Dalin Gong**^2^**, Guozheng Yang**^1^**, Shengqiu Feng**^1^**, Zhinan Mei**^1^**, De-Yu Xie**^3^**, Xuekui Wang**^1^**

**^1^** College of Plant Science and Technology, Huazhong Agricultural University, Wuhan, China.

**^2^** China Resources Sanjiu （Huangshi） Pharmaceutical Co., Ltd., Huangshi 435000, Hubei, China.

**^3^** Department of Plant and Microbial Biology, North Carolina State University, Raleigh, NC 27695, USA

Author for correspondence:

Xuekui Wang

E-mail: wang-xuekui@mail.hzau.edu.cn.

**Fig. S1.** Length distribution of PacBio long reads produced from the *T.sagittata* sample

**Fig. S2**. Genome assembly flowchart demonstrating assembly polishing and data integration.

**Fig. S3.** The Hi-C heatmap of *T. sagittata* genome assembly.

**Fig. S4.** The gene annotation methods of *T. sagittata* genome.

**Supplementary Figures**

**
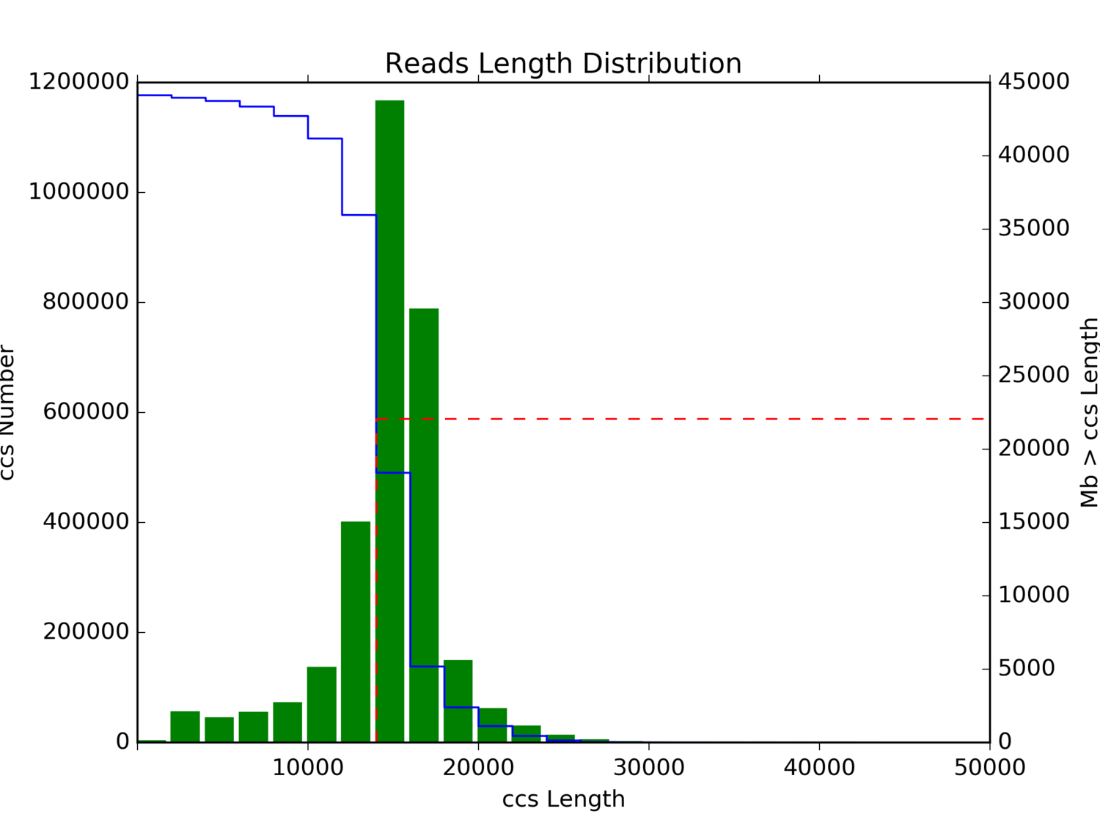
**

**Fig. S1.** Length distribution of PacBio long reads produced from the *T.sagittata* sample.


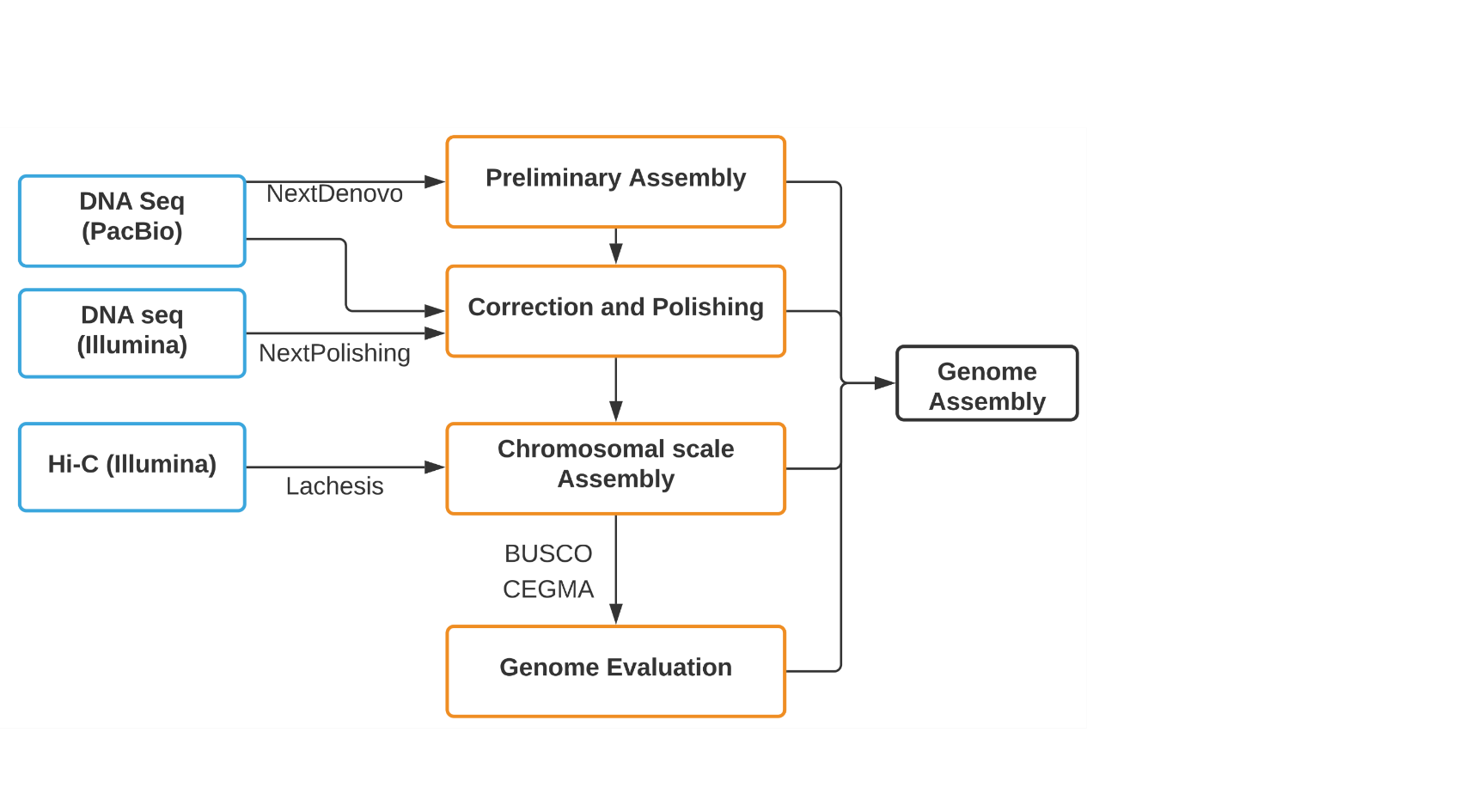


**Fig. S2**. Genome assembly flowchart demonstrating assembly polishing and data integration.


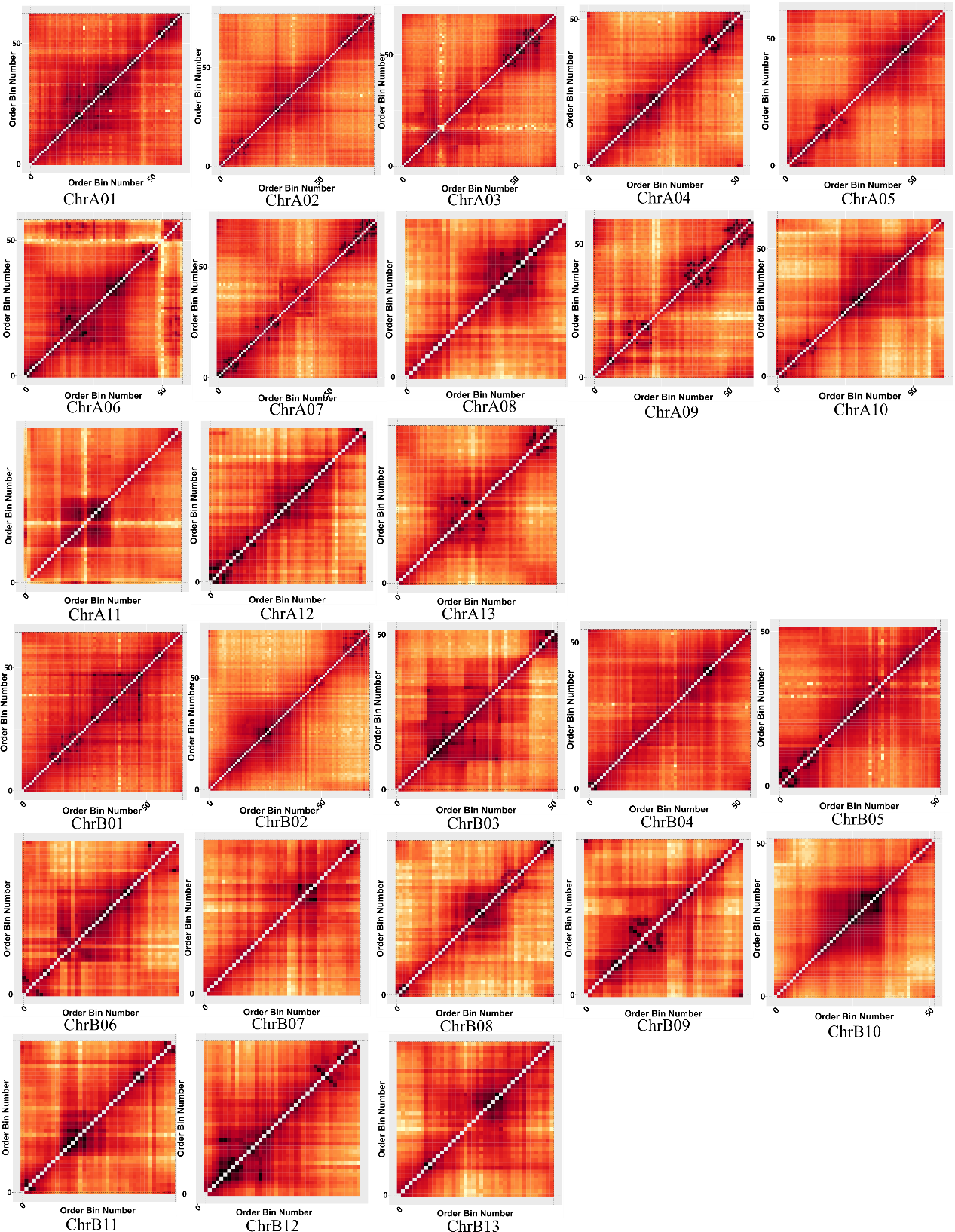


**Fig. S3.** The Hi-C heatmap of *T. sagittata* genome assembly.

**
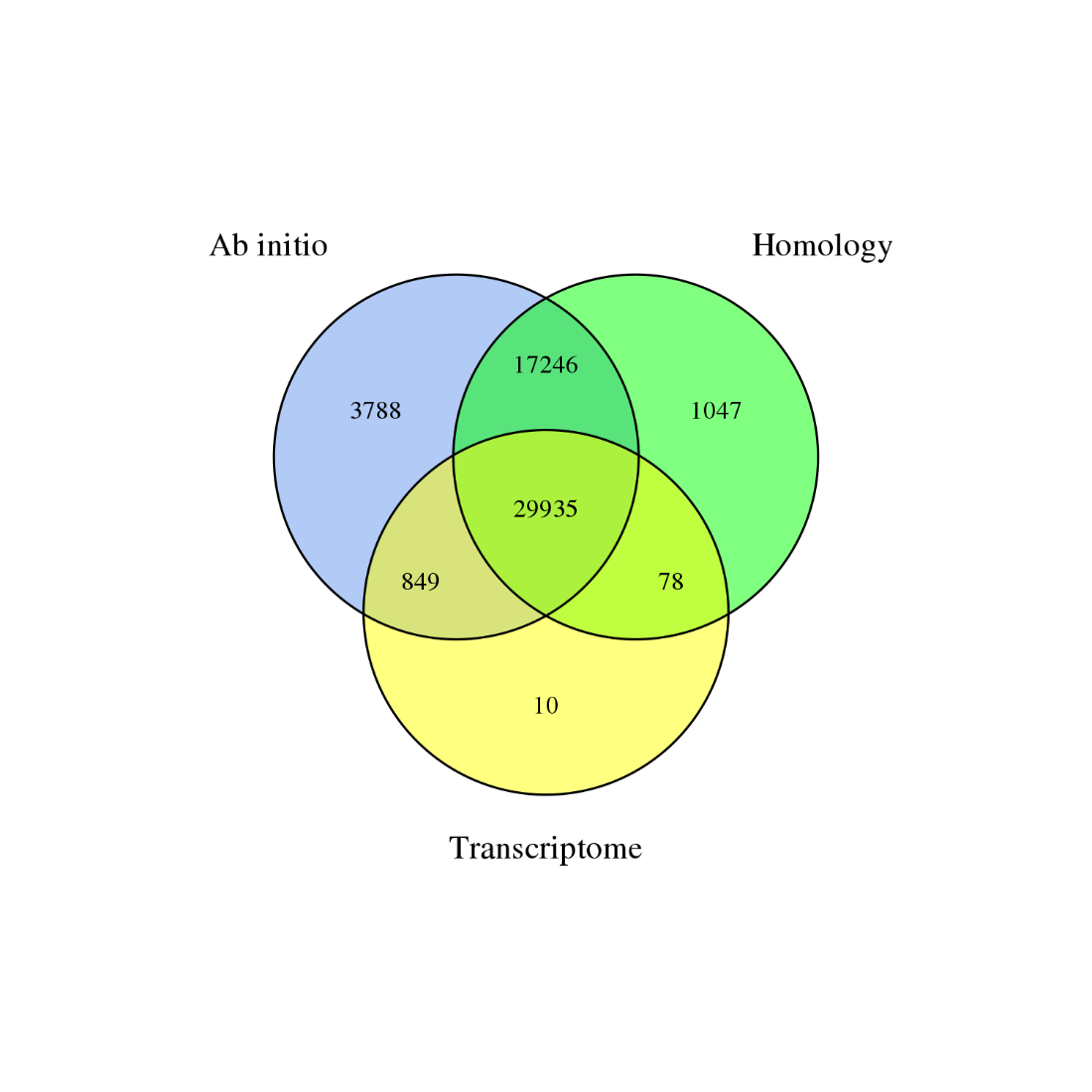
**

**Fig. S4.** The gene annotation methods of *T. sagittata* genome.
