# Supplementary material for: Chromosome-scale genome assembly of medicinal plant Tinospora sagittata (Oliv.) Gagnep. from the Menispermaceae family
Source: Sci Data. 2024 Jun 12;11:610. doi: 10.1038/s41597-024-03315-y (PMC11169364; doi:10.1038/s41597-024-03315-y)
Supplement: Supplementary file 3 — Supplementary Tables [file 41597_2024_3315_MOESM3_ESM.docx]

**Supporting information**

**Title:** **Chromosome-scale genome assembly of medicinal plant *Tinospora sagittata* (Oliv.) Gagnep. from the Menispermaceae family**

Mohammad Murtaza Alami**^1^**, Shaohua Shu**^1^**, Sanbo Liu**^2^**, Zhen Ouyang**^1^**, Yipeng Zhang**^1^**, Meijia Lv**^1^**, Yonghui Sang**^1^**, Dalin Gong**^2^**, Guozheng Yang**^1^**, Shengqiu Feng**^1^**, Zhinan Mei**^1^**, De-Yu Xie**^3^**, Xuekui Wang**^1^**

**^1^** College of Plant Science and Technology, Huazhong Agricultural University, Wuhan, China.

**^2^** China Resources Sanjiu （Huangshi） Pharmaceutical Co., Ltd., Huangshi 435000, Hubei, China.

**^3^** Department of Plant and Microbial Biology, North Carolina State University, Raleigh, NC 27695, USA

Author for correspondence:

Xuekui Wang

E-mail: wang-xuekui@mail.hzau.edu.cn.

**Table S1.** Sequencing data statistics of *T. sagittata*.

**Table S2.** Statistics of sequencing results.

**Table S3.** Reads length distribution statistics

**Table S4.** Coverage statistics of *T. sagittata* genome.

**Table S5.** Statistics on the quantity of each type of Hi-C sequencing data.

**Table S6.** Hi-C assembly data statistics table

**Table S7.** Primary statistical results of gene structure prediction of *T. sagittata* genome.

**Table S8.** The statistical results of gene function annotation of *T. sagittata* genome.

**Table S9.** Non-coding RNA statistical results in *T. sagittata* genome.

**Table S10.** Pseudogene prediction results in *T. sagittata* genome.

**Table S11.** Motif annotation information statistics of *T. sagittata* genome.

**Table. S12.** Statistics on data mapping of RNA sequencing.

**Table S13.** Repeat contents in the *T. sagittata* genome.

**Table S14.** Statistics of tandem repeats in *T. sagittata* genome.

**Table S1.** Sequencing data statistics of *T. sagittata*.

| **Pair-end libraries** | **Insert size** | **Total data (Gb)** | **Reads length (bp)** | **Sequence coverage (X)** |
| --- | --- | --- | --- | --- |
| Illumina reads | 350bp | 11.25 | 150 | 87.99 |
| PacBio reads | 60kb | 43 | - | 31.29 |
| Hi-C | 300-700bp | 2.33 |  |  |
| Total | - |  |  |  |

**Table S2.** Statistics of sequencing results.

| **Data Type** | **Reads number** | **Reads base** | **Reads Length N50** | **Reads Length Mean** | **Reads Length Max** |
| --- | --- | --- | --- | --- | --- |
| CCS | 2,589,389 | 40,662,123,998 | 15,761 | 15,703 | 45,967 |

**Table S3.** Reads length distribution statistics

| **Length** | **Total number** | **Total length** | **Average length** |
| --- | --- | --- | --- |
| 1~2000 | 383 | 572,822 | 1495.62 |
| 2000~4000 | 473 | 1,356,726 | 2868.34 |
| 4000~6000 | 273 | 1,386,209 | 5077.69 |
| 6000~8000 | 446 | 3,180,167 | 7130.42 |
| 8000~10000 | 716 | 6,498,223 | 9075.73 |
| 10000~12000 | 70,474 | 823,691,775 | 11687.88 |
| 12000~14000 | 354,980 | 4,632,843,843 | 13051.00 |
| 14000~16000 | 1,139,610 | 17,179,899,710 | 15075.24 |
| 16000~18000 | 773,668 | 12,979,214,628 | 16776.21 |
| 18000~ | 248,366 | 5,033,479,895 | 20266.38 |

**Table S4.** Coverage statistics of *T. sagittata* genome.

| Reads | Mapping rate (%) | 93.10 % |
| --- | --- | --- |

Average sequence depth: The average depth of each base on the genome covered by reads; Coverage: The proportion of genomes covered by reads.

**Table S5.** Statistics on the quantity of each type of Hi-C sequencing data.

| **Library** | **Type** | **Number** | **Ratio (%)** |
| --- | --- | --- | --- |
| Unknown AJ289-12H0001 | Unique Paired Alignments | 572,786,485 | 100 |
|  | Valid Interaction Pairs | 399,527,604 | 69.75 |
|  | Dangling End Pairs | 132475016 | 23.13 |
|  | Re-ligation Pairs | 6,957,566 | 1.21 |
|  | Self-cycle Pairs | 2,123,349 | 0.37 |
|  | Dumped Pairs | 50,447,641 | 8.81 |

**Table S6.** Hi-C assembly data statistics table

| **Chromosomes** | **Cluster Number** | **Cluster Length** | **Order Number** | **Order Length** |
| --- | --- | --- | --- | --- |
| ChrA01 | 96 | 53,081,736 | 21 | 50,497,308 |
| ChrB01 | 214 | 59,849,774 | 13 | 52,114,721 |
| ChrA02 | 67 | 64,078,053 | 35 | 63,024,682 |
| ChrB02 | 240 | 66,054,229 | 25 | 58,280,059 |
| ChrA03 | 41 | 56,258,608 | 22 | 55,640,768 |
| ChrB03 | 24 | 42,603,168 | 8 | 42,066,507 |
| ChrA04 | 22 | 43,470,943 | 14 | 43,050,440 |
| ChrB04 | 68 | 46,925,827 | 11 | 44,792,239 |
| ChrA05 | 75 | 51,378,322 | 44 | 50,323,249 |
| ChrB05 | 45 | 42,843,191 | 11 | 41,633,404 |
| ChrA06 | 55 | 47,939,945 | 22 | 46,634,346 |
| ChrB06 | 52 | 38,013,135 | 11 | 36,480,952 |
| ChrA07 | 49 | 58,134,879 | 36 | 57,734,895 |
| ChrB07 | 12 | 35,298,101 | 5 | 35,036,567 |
| ChrA08 | 7 | 32,100,129 | 1 | 31,913,866 |
| ChrB08 | 26 | 38,938,090 | 12 | 38,461,319 |
| ChrA09 | 33 | 48,425,503 | 24 | 48,118,239 |
| ChrB09 | 100 | 38,456,805 | 12 | 33,948,592 |
| ChrA10 | 63 | 51,667,667 | 22 | 50,254,553 |
| ChrB10 | 290 | 53,356,310 | 9 | 41,821,712 |
| ChrA11 | 42 | 44,237,293 | 6 | 38,281,874 |
| ChrB11 | 25 | 32,821,629 | 10 | 32,256,456 |
| ChrA12 | 157 | 41,724,543 | 26 | 36,905,758 |
| ChrB12 | 58 | 35,361,937 | 18 | 32,712,084 |
| ChrA13 | 29 | 38,635,237 | 14 | 38,135,351 |
| ChrB13 | 16 | 34,610,282 | 7 | 32,276,434 |
| Total (Ratio %) | 1906(42.52) | 1196265336(92.05) | 439(23.03) | 1132396375(94.66) |

**Table S7.** Primary statistical results of gene structure prediction of *T. sagittata* genome.

| **Gene set** | | **Number** | **CDS + intron length (bp)** | **Average CDS length (bp)** | **Average exon length (bp)** | **Average intron length (bp)** | **Average exons per gene** |
| --- | --- | --- | --- | --- | --- | --- | --- |
| *De novo* | Augustus | 47,402 |  |  |  |  |  |
|  | SNAP | 99,987 |  |  |  |  |  |
| Homolog | *A. coerulea* | 48,978 | 88,406,260 | 1226.16 | 1550.37 | 2011.09 | 5.24 |
|  | *A. thaliana* | 47,840 | 60,260,578 | 1219.2 | 1481.4 | 723.04 | 5.31 |
|  | *C. Chinensis* | 53,139 | 142,726,621 | 964.01 | 1141.54 | 2425.64 | 4.86 |
|  | *M. cordata* | 50,215 | 81,601,254 | 1258.67 | 1258.67 | 2465.55 | 5.3 |
|  | *T. sagittata* | - | 328,498,623 | 1360.42 | 1529.1 | 4674.48 | 5.52 |
| RNA-seq | Cufflinks | 36,805 |  |  |  |  |  |
|  | PASA | 21,315 |  |  |  |  |  |
| EVM |  | 52,953 |  |  |  |  |  |

**Table S8.** The statistical results of gene function annotation of *T. sagittata* genome.

| **Database** | **Annotated Number** | **Annotated Ratio%** |
| --- | --- | --- |
| GO | 43,905 | 82.91 |
| KEGG | 39,827 | 75.21 |
| KOG | 28,568 | 53.95 |
| Pfam | 45,633 | 86.18 |
| Swissprot | 43,390 | 81.94 |
| TrEMBL | 51,782 | 97.79 |
| eggNOG | 44,685 | 84.39 |
| NR | 51,317 | 96.91 |
| All | 51,855 | 97.93 |

**Table S9.** Non-coding RNA statistical results in *T. sagittata* genome.

| **rRNA number** | **tRNA number** | **miRNA number** | **snRNA number** | **snoRNA number** |
| --- | --- | --- | --- | --- |
| 13,014 | 9,624 | 292 | 350 | 227 |

**Table S10.** Pseudogene prediction results in *T. sagittata* genome.

| **Pseudogene** | **Stat** |
| --- | --- |
| Total Number | 287 |
| Total length | 1,189,507 |
| Average Length | 4144.62 |

**Table S11.** Motif annotation information statistics of *T. sagittata* genome.

| **Type** | **Stat** |
| --- | --- |
| motif | 2,429 |
| domain | 58,383 |

**Table. S12.** Statistics on data mapping of RNA sequencing.

| **Sample** | **Total Reads** | **Mapped Reads** | **Uniq Mapped Reads** | **Multiple Map Reads** | **Reads Map to '+'** | **Reads Map to '-'** |
| --- | --- | --- | --- | --- | --- | --- |
| L1 | 47,025,808 | 42,358,801 (90.08%) | 26,459,278 (56.27%) | 15,899,523 (33.81%) | 33,560,810 (71.37%) | 33,458,414 (71.15%) |
| L2 | 43,106,980 | 38,781,801 (89.97%) | 24,030,138 (55.75%) | 14,751,663 (34.22%) | 30,699,307 (71.22%) | 30,616,970 (71.03%) |
| L3 | 49,054,218 | 43,933,300 (89.56%) | 27,251,928 (55.55%) | 16,681,372 (34.01%) | 34,776,001 (70.89%) | 34,694,389 (70.73%) |
| R1 | 45,806,622 | 41,077,241 (89.68%) | 26,621,960 (58.12%) | 14,455,281 (31.56%) | 31,768,596 (69.35%) | 31,652,673 (69.10%) |
| R2 | 48,046,154 | 42,813,399 (89.11%) | 27,978,275 (58.23%) | 14,835,124 (30.88%) | 32,602,588 (67.86%) | 32,433,312 (67.50%) |
| R3 | 45,306,370 | 40,807,309 (90.07%) | 26,957,893 (59.50%) | 13,849,416 (30.57%) | 30,884,873 (68.17%) | 30,750,792 (67.87%) |
| S1 | 44,999,746 | 40,963,712 (91.03%) | 26,313,004 (58.47%) | 14,650,708 (32.56%) | 31,833,576 (70.74%) | 31,737,388 (70.53%) |
| S2 | 44,802,498 | 40,896,586 (91.28%) | 25,889,272 (57.79%) | 15,007,314 (33.50%) | 32,218,213 (71.91%) | 32,124,413 (71.70%) |
| S3 | 48,536,730 | 43,815,224 (90.27%) | 28,281,023 (58.27%) | 15,534,201 (32.01%) | 33,908,677 (69.86%) | 33,807,567 (69.65%) |
| T1 | 53,341,766 | 50,118,138 (93.96%) | 31,068,787 (58.24%) | 19,049,351 (35.71%) | 39,401,461 (73.87%) | 39,304,029 (73.68%) |
| T2 | 46,055,690 | 41,563,402 (90.25%) | 26,153,286 (56.79%) | 15,410,116 (33.46%) | 32,155,276 (69.82%) | 31,645,069 (68.71%) |
| T3 | 42,515,684 | 38,534,328 (90.64%) | 24,474,847 (57.57%) | 14,059,481 (33.07%) | 29,534,327 (69.47%) | 29,227,921 (68.75%) |

**Table S13.** Repeat contents in the *T. sagittata* genome.

| **Type** | **Number** | **Length** | **Rate (%)** |
| --- | --- | --- | --- |
| Classes: Retroelement | 843,195 | 586,585,186 | 45.13 |
| ClassI/DIRS | 1,258 | 370,767 | 0.03 |
| Classes / LINE | 124,296 | 37,981,420 | 2.92 |
| ClassI/LTR/Cassandra | 313 | 19,892 | 0 |
| ClassI/LTR/Caulimovirus | 4,018 | 4,144,415 | 0.32 |
| Classes / LTR / Copy | 211,805 | 186,958,708 | 14.39 |
| ClassI/LTR/ERV | 16,558 | 2,811,165 | 0.22 |
| ClassI/LTR/Gypsy | 239,772 | 241,091,501 | 18.55 |
| ClassI / LTR / Pao | 1,900 | 692,839 | 0.05 |
| ClassI/LTR/Unknown | 218,504 | 108,154,374 | 8.32 |
| ClassI/LTR/Viper | 86 | 11,026 | 0 |
| Classes / SINE | 24,685 | 4,349,079 | 0.33 |
| ClassII:DNA transposon | 275,809 | 85,622,126 | 6.59 |
| ClassII/Academ | 82 | 4,521 | 0 |
| ClassII / CACTA | 25,684 | 4,676,780 | 0.36 |
| ClassII/Crypton | 1,480 | 96,449 | 0.01 |
| ClassII/Given | 1,545 | 1,050,626 | 0.08 |
| ClassII/Ginger | 479 | 29,033 | 0 |
| ClassII/Helitron | 3,831 | 3,982,398 | 0.31 |
| ClassII/IS3EU | 471 | 27,746 | 0 |
| ClassII/Kolobok | 4,274 | 789,674 | 0.06 |
| ClassII / MITE | 391 | 96,661 | 0.01 |
| ClassII / Maverick | 1,088 | 63,981 | 0 |
| ClassII / Merlin | 247 | 14,106 | 0 |
| ClassII/Mutator | 15,784 | 8,250,188 | 0.63 |
| ClassII/Novosib | 657 | 37,768 | 0 |
| ClassII / P | 765 | 43,344 | 0 |
| ClassII/PIF-Harbinger | 4,469 | 596,859 | 0.05 |
| ClassII/PiggyBac | 1,363 | 131,007 | 0.01 |
| ClassII / Sola | 404 | 28,498 | 0 |
| ClassII/Tc1-Mariner | 8,751 | 1,246,331 | 0.1 |
| ClassII/Unknown | 160,605 | 54,802,998 | 4.22 |
| ClassII / Zator | 77 | 4,016 | 0 |
| ClassII/Zisupton | 873 | 55,788 | 0 |
| ClassII/hAT | 42,489 | 9,593,354 | 0.74 |
| Total | 1,119,004 | 672,207,312 | 51.72 |

**Table S14.** Statistics of tandem repeats in *T. sagittata* genome.

| **Type** | **Number** | **Length** | **Rate (%)** |
| --- | --- | --- | --- |
| Microsatellite (1-9 bp units) | 617,535 | 11,085,738 | 0.85 |
| Minisatellite (10-99 bp units) | 257,499 | 25,158,981 | 1.94 |
| Satellite (>=100 bp units) | 29,094 | 54,726,219 | 4.21 |
| Total | 904,128 | 90,970,938 | 7 |

Type: type of repetitive sequence; Number: number of repetitive sequences obtained; Length: total length of predicted repetitive sequences; Rate (%): the proportion of repetitive sequences in the total genome.
